# Letter from Mr. A. Clark, Pen Yan, Nov. 16th, 1839

**Published:** 1839-12

**Authors:** 


*Letter ftom Mr. A. Clark, Pen Yan, Nov.* 16*th*, 1839.

Messrs. Editors,

Having received and examined with some attention, and much pleasure and profit the first three numbers of your Journal, I beg leave to express my entire satisfaction with the general character of the work.

One design of the Journal, as stated by yourselves, and highly recommended by one of your correspondents, is to boldly expose "the arts of quackery." This, together with every honest man, I most cordially approve. But I am not of those who are eager to brand every improvement in dental science, and every departure from the beaten track however useful, with this opprobrious epithet.

The amalgam of mercury, as a filling for carious teeth, is called by one of your correspondents, an "execrable material." It may be so, and those who use it may be guilty of "dishonesty" and quackery. I do not pretend *now* to determine the point; I take the place of an inquirer for truth.

How does it appear that combinations of mercury, when employed as above stated, are necessarily destructive to the teeth? Does the external surface of the filling corrode, vitiate the saliva, and thus act externally on these organs ? Or does the mercury find its way into the stomach, and act through its influence on this organ ? Or does it operate chemically on the surface of the cavity in contact with it ? Or are these absorbents in the living teeth that introduce the mercury into their substance, analagous to its absorption when applied under certain cirmcumstances to the skin ? Does this article, the use of which is so much abused by quacks of one class, and so bitterly denounced by those of another, act upon the teeth, when used in combination as a filling in any or all of these modes ? What, gentlemen, *is* the modus operandi of the thing ? Will you, or some of your correspondents inform us ?

Whatever be the mode of injury, is it always speedily apparent, or is it slow, effecting imperceptibly, at first, its destructive work ? And if the latter, about how long must we wait to have an experiment satisfactory ?

Argument based on indisputable facts is desired; the dictum of no man, though gray in his profession, will be deemed sufficient.

The scepticism on this subject, these queries may seem to imply, may subject their author to the imputation of ignorance. Well, be it so, many a man has died a fool because he would not seek for knowledge, lest he expose his ignorance.

